# Periosteal medial femoral condyle free flap for metacarpal nonunion

**DOI:** 10.1002/micr.30826

**Published:** 2021-10-11

**Authors:** Thierry Christen, Swenn M. Krähenbühl, Camillo T. Müller, Sébastien Durand

**Affiliations:** ^1^ Department of Plastic and Hand Surgery Centre Hospitalier Universitaire Vaudois Lausanne Switzerland

## Abstract

**Background:**

Metacarpal nonunion is a rare condition. The osteogenic capacity of periosteal free flap was investigated in five patients with metacarpal nonunion and impaired bone vascularization.

**Patients and methods:**

Surgery was performed between 64 and 499 days after the initial bone osteosynthesis. The average age was 27.6 (range 16–32) years. Nonunion was caused by excessive periosteal removal in two patients, extensive open trauma in three. Four nonunions were diaphyseal, one metaphyseal. A periosteal medial femoral condyle free flap was raised on the descending genicular artery for four patients, the superomedial genicular artery for one. After osteosynthesis with a plate, the flap was wrapped around the metacarpal, overlapping the bone proximally and distally. The recipient vessel were the dorsal branch of the radial artery and a vena comitans in the anatomical snuffbox in four patients, at the base of the first webspace in one.

**Results:**

The flap size ranged from 5 × 3.5 cm to 8 × 4 cm. No postoperative complication occurred. Radiological bone union was achieved 3 to 8 months after surgery. One patient had a full range of motion, one a slight extension lag of the proximal interphalangeal joint, two moderate joint stiffness of the proximal interphalangeal or metacarpophalangeal joint (one requiring plate removal and extensor tenolysis), one severe stiffness that allowed using a hook grip which was the aim of the surgery.

**Conclusion:**

In case of metacarpal nonunion with impaired bone vascularization, the periosteal medial femoral condyle free flap provides an effective and biomimetic approach to bone healing.

## 1 INTRODUCTION

Metacarpal fractures account for ~42% of all hand fractures (Feehan & Sheps, 2006) however, metacarpal nonunion is a rare condition (Ring, 2006). Through the impairment of bone vascularization, extensive open trauma, or excessive periosteum removal during surgery are known sources of nonunion (Choudry et al., 2008). The absence of periosteum decreases the ability of callus formation (Gajendran et al., 2015). The rate of successful nonunion management after traditional means of internal fixation and bone grafting ranges from 70% to 92% in the absence of major skeletal loss (Ring, 2006). In cases of poorly vascularized bone beds and open comminuted fractures, achieving bone union may be compromised and further surgery necessary in 30% (Choudry et al., 2008).

The medial femoral condyle flap was initially described as a pedicled periosteal or corticoperiosteal flap for skeletal reconstruction of the lower limb (Hertel & Masquelet, 1989). It was used as a free corticoperiosteal flap to treat recalcitrant upper limb nonunions, such as clavicle, humerus, and scaphoid (Fuchs et al., 2005; Jones Jr. et al., 2008; Muramatsu et al., 2003). Effectiveness of the technique has been shown by numerous experimental and clinical studies over the past decades confirming the osteogenic properties of periosteum (Camilli & Penteado, 1994; Ortak et al., 2005). Previous reports appear to demonstrate a similar effect on ossification abilities between corticoperiosteal and periosteal‐only flaps (Vegas et al., 2012). The aim of this study was to investigate the osteogenic capacity, the advantages, and the outcome of free periosteal‐only medial femoral condyle (PMFC) flaps in patients with metacarpal nonunion with impaired bone vascularization.

## 2 PATIENTS AND METHODS

Written consent was obtained from all patients, and the procedures were performed in line with the Helsinki Declaration of 1975 (recommendations guiding medical doctors in biomedical research involving human subjects).

Patients underwent surgery between 64 and 499 days after the initial trauma. Average age was 27.6 years‐old (range: 16–32). The cause of the nonunion was excessive periosteum removal in two cases, extensive open trauma in three. The nonunion was diaphyseal in four patients, metaphyseal in one. Two patients were smokers and were asked to stop smoking before the surgery. The patient characteristics are shown in Table 1.

**TABLE 1:** Patient characteristics, treatment and outcome

Patient	Age (years)	Sex	Fracture type	Trauma	Initial treatment	Delay before definitive surgery	Source of devascularization	Flap size (cm)	Bone fixation	Additional cancellous bone graft	Complications	Time to union (months)	Clinical result
1	29	M	Diaphyseal transverse, simple	Closed, blunt	ORIF w/plate	254	Excessive periosteum removal	‐	Plate	None	None	3	Full ROM
2	30	F	Diaphyseal, multifragmentary	Open, blast	ORIF w/K‐wire and cement	264	Extensive open trauma	5 × 3.5	Plate	Autograft	None	7	Stable fifth ray allowing hook grip
3	31	M	Diaphyseal, multifragmentary	Open, gunshot	ORIF w/K‐wire	64	Extensive open trauma, infection, bone loss	8 × 4	Plate	Autograft	None	5	Slight extension lag (15°) of the PIP joint
4	16	M	Metaphyseal, multifragmentary	Open, saw	ORIF w/plate	299	Extensive open trauma	‐	Plate	Autograft	None	7	Limited flexion (60°) of the PIP joint
5	32	F	Diaphyseal, multifragmentary	Closed, blunt	ORIF w/plate	499	Excessive periosteum removal	7 × 4	Plate	Allograft	None	8	MP joint stiffness in extension

### 2.1 Surgical technique

The PMFC flap was harvested through an incision on the distal third of the medial thigh. The vastus medialis fascia was incised posteriorly, allowing exposition of the medial femoral condyle and its periosteal blood supply (Kazmers et al., 2017). The dominant vessel, the descending genicular artery or the superomedial genicular artery, was identified and dissected up to its origin. The periosteal flap was harvested with its pedicle and transferred to the hand. The nonunion site was prepared after removal of hardware, only minimal bone resection was done. The recipient vessels were identified. Metacarpal osteosynthesis was performed with a plate. Cancellous bone graft, either autogenic or allogenic, was inserted between the bone stumps in the presence of bone loss. The periosteum was wrapped around the metacarpal shaft and the plate at the level of the defect. End‐to‐side anastomosis was performed to the radial artery and end‐to‐end to a *vena comitans*. Skin was sutured after ensuring perfusion of the flap.

Patients were discharged the day after surgery. Aspirin (100 mg) was given once a day during 1 month. Color Doppler ultrasonography assessed flap perfusion before discharge (day 1) and at suture removal (day 10). Bone union was confirmed by x‐rays and/or CT‐scan.

## 3 RESULTS

In all patients, the metacarpal was macroscopically devascularized, ivory white, with no periosteum remaining in vicinity of the nonunion. No bone bleeding was apparent after debridement of the nonunion site. Patient 3 had bone loss to the ballistic nature of the trauma.

The flap was harvested on the descending genicular artery for four patients, on the superomedial genicular artery for one patient. The size of the flap ranged from 5 × 3.5 cm to 8 × 4 cm. Postoperative course was uneventful, no complications occurred. In all cases, bone union was obtained with a mean time of 6 months (range 3–8). Patient 1 had complete active range of motion. Patient 2 showed severe stiffness of the fifth ray (the four other rays were missing) but recovered a painless hook grip which was the aim of the surgery. In Patient 3, the plate was removed and an extensor tenolysis performed to reach full active range of motion. Patient 4 was satisfied with the result and did not consider further surgery even though flexion of the PIP joint was limited to 60°. Flexion of the MP joint was limited to 25° in Patient 5 because of adhesions between the extensor tendon and the plate. Tenolysis and plate removal are scheduled. The same patient was not able to refrain from smoking although she agreed preoperatively to do so. Her time to bone union was the longest of this series. The results appear in Table 1.

### 3.1 Case report

**FIGURE 1:**
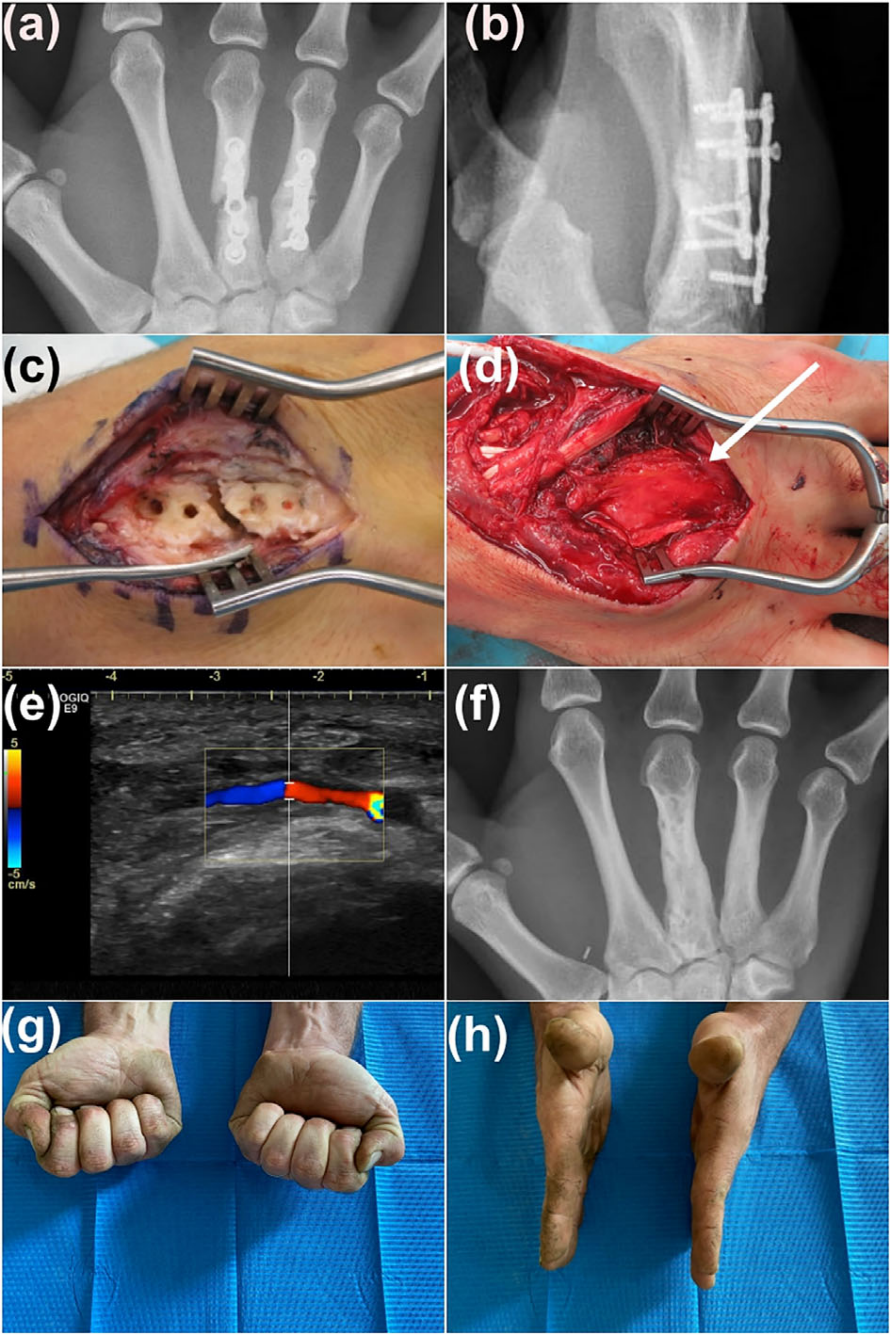
Illustration of Patient 1. (a) and (b) X‐rays showing nonunion of the third metacarpal, the fourth metacarpal fracture has united; (c) Devascularized aspect of the fourth metacarpal due to excessive periosteal stripping; (d) periosteal flap (arrow) wrapped around the metacarpal and plate, sutured to itself, isolating the nonunion in a “regeneration chamber”; (e) color Doppler ultrasound showing patency of the arterial anastomosis 10 days after surgery; (f) X‐ray confirming bone union, the plate has been removed; (g) and (h) complete range of motion after 5 years

A 29 year‐old male landscaper sustained a transverse diaphyseal fracture of the right third and fourth metacarpals following blunt trauma. Both fractures were plated. Bone union was achieved after 2 months on the fourth ray while the third metacarpal developed painful nonunion (Figure 1a). Eight months after the initial surgery, both plates were removed. The third metacarpal appeared macroscopically devascularized, no periosteum remained on its surface (Figure 1b). A new plate stabilized the third metacarpal and cancellous bone graft taken from the distal radius was placed between the bone stumps. A PMFC free flap was harvested from the ipsilateral knee with the descending genicular artery and two vena comitans. It was wrapped circumferentially around the metacarpal and plate, overlapping the bone on either side (Figure 1c). Microvascular anastomosis were performed on the dorsal branch of the radial artery (end‐to‐side) and a vena comitans (end‐to‐end). The flap showed punctate bleeding confirming adequate blood flow. The wrist was immobilized in a splint for 3 weeks, the fingers were free to move. A color Doppler examination showed adequate flap perfusion on the first and tenth postoperative days (Figure 1d). Three months after the surgery, bone union was obtained and the patient was able to resume his professional occupation (Figure 1e). The range of motion was complete and the patient reported no pain at the level of the hand or knee (Figure 1f). He requested removal of the plate, which was done 7 months after the flap.

## 4 DISCUSSION

The majority of diaphyseal nonunions of the long bones can be successfully treated by osteosynthesis and bone grafting (Babhulkar et al., 2005). The main risk factors for developing a recurring nonunion are a poorly vascularized bone bed after previous infection, initially open and severely comminuted fracture, or internal fixation with extensive iatrogenic periosteum removal. In these cases, vascularized bone grafting might be justified (Doi & Sakai, 1994; Georgiadis et al., 1993; Gonzalez del Pino et al., 2004).

The PMFC flap should be considered as an alternative in cases of nonunion with or without segmental bone defect. It can be fashioned to any shape around the defect promoting conventional bone healing mechanisms with or without cancellous bone graft.

Several studies have assessed the use of periosteal flaps for nonunion management, mostly in children. Soldado et al. have shown excellent results using various free and pedicled periosteal flaps without bone graft in skeletally immature patients (Barrera‐Ochoa et al., 2018; Soldado et al., 2020). The same group also reported a favorable outcome in adult scaphoid nonunion management with a similar technique (Barrera‐Ochoa et al., 2020). We chose to use the PMFC for various reasons: its size is large enough to easily wrap around a metacarpal and bridge a potential bone gap; the donor site morbidity is low (Mehio et al., 2018); harvesting the flap poses no technical difficulty and the vessel size is rather large; no major artery needs to be sacrificed.

Our results demonstrate that adult periosteal flaps are able to promote bone growth on devascularized adult metacarpals. Ongoing smoking does not seem to prevent achieving bone union, nor does the absence of cancellous bone graft. The color Doppler imaging confirmed that the flaps were perfused at 10 days. We cannot exclude that the sturdy plating provided enough stability to allow by itself bone union however, this seems unlikely since bone devascularization was evident during surgery.

The periosteum is involved in all phases of the fracture repair process, providing cells for both osteogenesis and chondrogenesis, participating in intramembranous and endochondral ossification (Thompson et al., 2015). The free periosteal graft, when wrapped around the defect and sutured to itself, provides a regeneration chamber with the proper environment to stimulate physiological pathways to promote bone union. This enables both direct and indirect ossification.

Indirect bone healing with endochondral ossification was the main process of bone formation in Patient 1 where the sole presence of a vascularized periosteum was sufficient to allow ossification. Indirect bone healing follows a specific biological pathway involving an acute inflammatory response with production and release of pro‐inflammatory cytokines. These allow the recruitment of inflammatory cells that promotes local angiogenesis and induce the osteogenic differentiation of mesenchymal stem cells into a primary fusiform cartilaginous callus (Marsell & Einhorn, 2011) (Patient 1).

Angiogenesis is critical to the direct intramembranous healing. The periosteum brings circulating growth factors, migrating bone cell precursors, and vascular endothelial cells in proximity to the nonunion. In this way, it provides the angiogenetic *niche* necessary for osteogenesis and bone consolidation (Gerstenfeld et al., 2003; Percival & Richtsmeier, 2013). This pathway allows for immediate regeneration of anatomical lamellar bone without remodeling steps as in Patients 2, 3, 4, and 5. Recently, authors have demonstrated that vascularized periosteal flaps promote and accelerate allograft‐host bone union in a rat model (Gallardo‐Calero et al., 2019).

Anatomical variations of the PMFC flap are frequent and should be known as they can have an impact on the pedicle length (5) when the inflowing artery is the superomedial genicular artery instead of the descending genicular artery. The maximal surface of the flap that can be raised without vascular compromise is 10 by 4 cm (Hertel & Masquelet, 1989; Sakai et al., 1991), large enough to manage any metacarpal nonunion.

Conventional orthopedic algorithm for the treatment of nonunion is composed of resection of nonunion, osteosynthesis, and bone grafting. This approach provides the shape and the structure but the physiological environment is missing. Therefore, it might seem insufficient particularly when vascularization of the bone is compromised. Enhancing local vascularization can be obtained with the induced membrane technique (Masquelet et al., 2000; Moris et al., 2016). However, this procedure requires two surgeries and does not provide a tissue specifically intended to allow bone formation. We propose a more biomimicry‐oriented approach, which achieved union in all five patients despite the lack of initial bone vascularization. In an era where microsurgery is widely available, PMFC flap could be a first‐line alternative in cases of poorly vascularized metacarpal nonunion.

## 5 CONCLUSION

The free PMFC flap appears to be a reliable technique to achieve bone union in cases of metacarpal nonunion with poorly vascularized bone.

## Data Availability

Data are available on request from the authors.
